# Methodologic and Logistic Issues in Conducting Longitudinal Birth Cohort Studies: Lessons Learned from the Centers for Children’s Environmental Health and Disease Prevention Research

**DOI:** 10.1289/ehp.7670

**Published:** 2005-06-24

**Authors:** Brenda Eskenazi, Eleanor A. Gladstone, Gertrud S. Berkowitz, Christina H. Drew, Elaine M. Faustman, Nina T. Holland, Bruce Lanphear, Stefanie J. Meisel, Frederica P. Perera, Virginia A. Rauh, Anne Sweeney, Robin M. Whyatt, Kimberly Yolton

**Affiliations:** 1 Center for Children’s Environmental Health Research, School of Public Health, University of California, Berkeley, California, USA; 2 Department of Community and Preventive Medicine, Mount Sinai School of Medicine, New York, New York, USA; 3 Center for Child Environmental Health Risks Research, Department of Environmental Health, University of Washington, Seattle, Washington, USA; 4 Children’s Environmental Health Center, Children’s Hospital Medical Center and University of Cincinnati College of Medicine, Department of Environmental Health, Cincinnati, Ohio, USA; 5 Columbia Center for Children’s Environmental Health, Mailman School of Public Health, Columbia University, New York, New York; 6 The Friends Children’s Environmental Health Center, University of Illinois, Urbana-Champaign, Illinois, USA

**Keywords:** biologic samples, biorepository, birth cohort, children, environmental health, ethics, growth, National Children’s Study, neurodevelopment, pregnancy

## Abstract

In anticipation of the National Children’s Study, lessons can be learned from the smaller birth cohort studies conducted by five Centers for Children’s Environmental Health and Disease Prevention Research funded by the National Institute of Environmental Health Sciences and the U.S. Environmental Protection Agency. The populations studied are diverse in ethnicity and social class and reside in urban and rural environments. Although almost all of the centers chose to enroll participants through medical care facilities, they had to develop independent staffs and structures because of the overburdened medical care system. Some of the lessons learned by the centers include the importance of continuous funding, building community partnerships to conduct culturally appropriate research, hiring bilingual and bicultural staff from the community, prioritizing research goals, developing biorepositories to ensure future utility of samples, instituting quality control procedures for all aspects of specimen and data collection, maintaining frequent contact with study participants, ensuring ethical conduct of the research in a changing medical-legal climate, and communicating results in a timely and appropriate manner to participants and the wider community. All centers underestimated the necessary start-up time, staff, and costs in conducting these birth cohort studies. Despite the logistical complexity and added expenses, all centers emphasize the importance of studying the impact of environmental exposures on those children most at risk, those living in minority and low-income communities. These centers present barriers encountered, solutions found, and considerations for future research, with the hope that the lessons learned can help inform the planning and conduct of the National Children’s Study.

Longitudinal birth cohort studies provide a rich source of information about antecedents of disease that originate in pregnancy or childhood. There have been two previous major longitudinal birth cohort studies in the United States: the Child Health and Development Studies (van den Berg et al. 1988) and the National Collaborative Perinatal Project (Niswander and Gordon 1972). Since these studies were conducted, > 40 years ago, science and research infrastructures have changed dramatically in the United States. These changes include but are not limited to advances in bio-markers and molecular and cellular biology, the use of computers in tracking and statistics, the increased difficulty of integrating research into routine clinical practice, the diversity and health disparity of the population, and growing complexities of medical-legal-ethical issues. The standards for quality research have risen considerably and with them the logistical complexities in conducting such research.

The purpose of this article is to outline the methods used by the five Centers for Children’s Environmental Health and Disease Prevention Research (Children’s Centers) that are conducting birth cohort studies. Three centers (University of California, Berkeley, Columbia University, and Mount Sinai School of Medicine) began their studies in 1998, and two centers (University of Cincinnati and University of Illinois) in 2000. All but two centers (Columbia and Mount Sinai), which had prior funding, started enrollment approximately 1 year after funding. Table 1 provides an overview of the five centers (also see Kimmel et al. 2005), each of which recruited racially/ethnically diverse and often low-income populations (Appalachian, Dominican, Hmong, Laotian, Mexican, African American, and Puerto Rican). Our centers worked closely with our respective communities to develop partnerships, strengthen community infrastructure, build trust, and conduct culturally appropriate research (Israel et al. 2005). We present here some of the barriers we faced and our solutions, with the hope that the lessons learned from our experience will assist in the planning and conduct of the National Children’s Study (2005).

## Recruitment and Enrollment

Table 2 shows the eligibility criteria and recruitment strategies for each of the studies. Four centers recruited pregnant women. Three centers enrolled women before their third trimester of pregnancy; one center (Columbia) enrolled women throughout pregnancy, and another (Illinois) recruited couples before and during pregnancy.

The center at Illinois attempted to screen all Hmong and Laotian families in the study catchment area for eligibility, using telephone directories as suggested by community leaders. Study workers contacted all people with Hmong or Laotian surnames listed in the directory to describe the study and determine eligibility. Home visits were scheduled with eligible, willing families and were repeated every 2 months. Pregnancy tests were performed at each visit, and couples were enrolled in the cohort study when the women became pregnant.

The other four centers recruited through multiple hospital or clinic sites. They attempted to recruit as many consecutive, eligible patients as possible. The center at Berkeley used clinic staff to screen women for eligibility; eligible women were shown a video about the study and, if interested, were referred to a study worker. Berkeley also enrolled fathers, with only half participating. The center at Cincinnati, with a Health Insurance Portability and Accountability Act (HIPAA 1996) waiver, received weekly information about new patients directly from the clinics. Eligible patients were sent a letter describing the study and a mail-in postcard to decline further contact; those who did not return the postcard were contacted by phone to set up an appointment. The study staff at Columbia recruited participants by approaching women in clinic waiting areas, and the Mount Sinai staff recruited women from a prenatal clinic and two private practices.

Eligibility and exclusion criteria varied. The study populations differed considerably with respect to race/ethnicity, socioeconomic status, and geographic setting (urban vs. rural). Only one center specifically recruited primiparas; the same center was unique in its enrollment of mothers < 18 years of age. Although enrolling minors was not a problem for this center, other centers chose not to include minors because of additional institutional review board (IRB) requirements. Three centers excluded infants from continued follow-up if prenatal specimens or data were not collected. One of these centers also excluded infants if they were high risk (< 32 weeks gestation, < 1,500 g, or having congenital malformations). Some centers determined eligibility by race (e.g., African American) or country of birth (e.g., Dominican), whereas others determined eligibility by language (e.g., Spanish or English speaking). A number of the centers required some stability in residence or in medical care (e.g., planning to deliver at the hospital where the study was based) to be eligible. One center required that the women had lived in the study area for at least a year before pregnancy and planned to remain for at least a year afterward.

### Overcoming barriers to recruitment and enrollment.

Participation rates for the studies ranged from 25 to 64%. All centers gathered demographic information on all eligible women, permitting later comparison of participants with eligible nonparticipants.

The most important barriers to participation, especially for working women, were the time required for each visit and the length of the follow-up period. Centers that recruited patients from clinic waiting areas found that even short waiting periods, especially in private practice offices, were a barrier. The one center using clinic staff for recruitment found that they were already overburdened and had little time for recruitment. Some centers also found that women were reluctant to enroll without their husband’s approval.

Many of the populations of interest in children’s environmental health studies are economically disadvantaged, undereducated, non-English-speaking, and distrustful of Western medicine and research. Many centers found that hiring study staff familiar with or from the target population was necessary for successful recruitment. Recruitment by or at clinics known by the community to respect patient confidentiality was particularly successful. In addition, response rates were improved by allowing potential participants time to discuss the study with their families before enrollment.

## Assessment Methods

The centers have used a variety of tools to gather information about their cohort study participants (Table 3). At multiple time points during and after pregnancy, mothers completed questionnaires that focused on demographics, medical history, and exposure information (Appendix 1). Illinois included prepregnancy baseline questionnaires and menstrual cycle tracking. Several centers also completed multiple home visits over the course of the study. Questionnaires and home visits were completed in intervals ranging from every 3 months to annually; home visits were usually conducted at the same time points as questionnaires. In addition, all centers conducted neurodevelopmental and growth assessments, and most collected information on medical conditions such as asthma.

### Growth, Development, and Other Health Outcomes

Most centers conducted neurodevelopmental assessments and growth assessment at numerous age points after birth (Dietrich et al. 2005). Various standardized neurodevelopmental assessment tools were used to assess the neonate, infant, and child. Two centers plan to collect school-based evaluations such as report cards and teacher ratings of classroom behavior. All centers used standardized anthropometric measurement protocols to measure height, weight, and head circumference at each contact point, some taking multiple measurements to reduce measurement error. Most centers used questionnaires and medical record review to obtain prenatal and child health information for respiratory disease (Eggleston et al. 2005) and other outcomes. Medical records were either abstracted for complete information or to confirm reported conditions. One center received all prenatal and delivery information on computer-ready forms from the participating hospital.

### Social Environment

As prescribed by the centers’ Request for Application, many of the participants in these studies were from marginalized, low-income communities. Hence, most centers assessed aspects of the children’s social environment (Appendix 1) expected to affect their health and development. These measurements were obtained from observation, face-to-face interviews, and/or direct child assessment. All centers gathered information about the home environment and household composition, including presence of the father. Almost all centers used the Home Observation for the Measurement of the Environment scale (Caldwell and Bradley 1984). Others included measures of maternal depression, social support, parenting and marital stress, and use of childcare services. Centers that included immigrant populations obtained information on immigration history and acculturation. Socioeconomic status was ascertained by all centers; besides measuring total income and income per person supported, a few centers determined overall material hardship, food security, and use of social services.

### Physical Environment

All centers assessed housing quality via questionnaire. In addition, three centers conducted home visits (see Table 3). Two centers used Global Positioning System (GPS) devices to determine the proximity of the home to services, pesticide applications, and high-crime or traffic areas (Gilliland et al. 2005). To assess the condition of the housing stock, centers either modified a measure developed by the U.S. Department of Housing and Urban Development (Jacobs et al. 2002) or developed their own instruments, which included visual assessments for molds/mildew, deteriorated paint, safety hazards, leaks, roach/rodent infestations, and other factors (Appendix 2). Because there are few validated tools to assess exposure to indoor pollutants, creating these materials was challenging. Home inspections themselves were time-consuming and required extensive training, in some cases provided by county housing inspectors. Several centers opted to visit homes multiple times to reassess household exposures, which may vary by season (Yiin et al. 2000) or change when families relocate.

During the home inspections, centers collected ambient measurements and samples, including wall moisture levels, mattress and floor dust samples, and air samples (Table 4).

Collecting environmental measurements often required the purchase of expensive, specialized collection equipment (e.g., air monitors) and a delay between home assessments to allow for cleaning of equipment. Standard practices for interpreting ambient measurements are not yet fully developed; for example, for most contaminants, it is unclear whether house dust concentration (micrograms per gram of dust) or loading (micrograms of surface area) is a better predictor of children’s exposure or body burden.

### Overcoming Barriers to Assessment

#### Delivery events.

In the immediate postpartum period, some centers conducted neonatal assessments and interviews with the mother and, in one center, with the father. In addition, all centers attempted to collect biologic specimens during this time period. Shortening post-delivery hospital stays in the United States left a limited window of opportunity to collect information and samples from mothers and neonates in the hospital. Although women remained in the hospital for 48 hr after delivery at most centers, discharge was occasionally earlier.

Although all centers anticipated quick notification of participants’ admission for delivery, this was often overlooked in the frenzy of labor and birth. All centers relied on both participating women and delivery ward staff for notification. At one center, mothers without home phones were initially given cell phones to call the research team, whereas at other centers women were given special tee shirts or socks to wear to the hospital so as to alert the delivery staff. Some centers provided lists of participants approaching their due date to the medical station, and many checked delivery ward logbooks on a daily basis. Despite these efforts, for most centers where enrollment has ended, a large proportion of women were not tracked at the time of delivery (> 25%). In addition, nighttime and weekend admissions required that center staff were available at all times, sometimes resulting in costly overtime hours. Those centers whose employees were already integrated into the clinical program had a somewhat easier time completing delivery events.

Cord blood samples were particularly difficult to obtain; collection rates at the five centers ranged from 40 to 85%. Most missed collections occurred when women’s delivery admissions were not reported to research staff, although additional samples were missed from high-risk children with emergency deliveries. At least one center collected data at a hospital that did not routinely collect cord blood samples; when it did, a method was used that could result in contamination with maternal blood. In another case, hospital staff were concerned about accidental needle sticks from the traditional venipuncture collection method; this center worked with the hospital to develop an acceptable alternative. The greatest collection rate was reported by the one center that involved physicians on the research team in collecting the samples.

Numerous difficulties were encountered in conducting neonatal assessments. Few tests are available to assess newborn behavior, and their predictive validity is not high. The Brazelton Neonatal Behavioral Assessment Scale requires substantial training, which is available in only a few locations (Lester and Tronick 2001). Trained evaluators who left projects could not be easily replaced, sometimes leaving gaps in cohort assessment.

Although most centers attempted to complete neonatal assessments during the post-delivery hospital stay, this was not always possible. Assessments could not occur too soon after delivery lest behavior be affected by delivery medications, and short hospital stays left little time to schedule assessments when the child was not sleeping or eating. In addition, it was difficult to find a quiet assessment room in some hospitals and interruptions by medical personnel were common. The effect of these obstacles was that assessments intended for the neonatal period were in many cases conducted several weeks after delivery; again, high-risk children who required extra neonatal care were the most difficult to assess in a timely manner. One center increased success with hospital assessments by conducting early morning assessments. Another center chose to assess the child twice, soon after delivery and again a month later.

#### Participant fatigue.

Recognizing that participation in a longitudinal study is demanding for families, all centers attempted to minimize inconvenience to participants. Centers aimed to optimize contact frequency such that attrition was prevented but participants were not overly bothered. Likewise, all centers designed their contacts to be as brief and efficient as possible. This was particularly challenging for neurodevelopmental assessments; every additional developmental domain assessed increased the risk that children would be excessively fatigued. Many centers found it necessary to narrow the focus of their research questions out of respect for participants’ time.

Centers used several strategies to prevent participant fatigue. Some centers used multiple workers to simultaneously collect information at each visit (e.g., separate assessments of mother and child). This sometimes required that staff were trained in multiple aspects of the study protocol, for example, phlebotomists who were trained to conduct interviews. Some centers found that participants preferred multiple short visits to one long visit, both for convenience and to prevent child fatigue. Weekend and evening assessments, although needed by working parents, pose a strain on staff. For lengthy and demanding assessments, centers sometimes provided snacks and childcare to participants.

Many centers found it critical to use flexible assessment tools that could accommodate necessary protocol changes. Shortened versions of in-person questionnaires were found useful for telephone interviews when participants were not available to meet face to face. Some centers found it helpful to organize neurobehavioral assessments with the most important items first, minimizing data loss when children fatigued. Most centers also developed qualitative assessments that allowed study staff to document participants’ level of fatigue, cooperation, and attention and to record any changes made to the usual study protocol.

#### Distractions.

Distractions during interviews and assessments posed a challenge for all centers. Conducting assessments in the home was nearly impossible, especially for participants in crowded living conditions; thus, the provision of a standardized testing facility was essential. Minimizing distractions to children during neurobehavioral assessments was particularly challenging. For children > 12 months of age, it was desirable to assess the child separately from the mother to reduce interference; this, however, required additional time for the tester to build rapport with the child. Siblings were also a source of distraction during assessments. Centers accommodated siblings by providing on-site childcare, giving reimbursements for off-site childcare, and/or using videos or games to busy these children; however, most agreed that on-site childcare with dedicated space and personnel would be preferable to these arrangements. Finally, centers helped minimize disruptions to child assessments by scheduling bathroom and snack breaks.

A related issue was participant privacy. For one center, fathers frequently wanted to be present at maternal interviews. Because of concerns that the mothers might not answer personal questions honestly in their presence, partners were not permitted to attend.

#### Quality control of assessments and interviews.

All centers emphasized the importance of proper staff training. However, although all centers considered pilot testing extremely important, most expressed frustration that time, cost, and the need for prior IRB approval often hindered adequate pilot testing with noncohort participants.

Most centers instituted clear quality control protocols, particularly for the neurodevelopmental assessments. These included direct observations or review of videotapes by the other evaluators and lead psychologists. However, insufficient time and resources caused many centers with clear quality control protocols to fall short of what they considered appropriate quality control (e.g., taping and reviewing ~10% of assessments). Some centers expressed concern about inter-rater differences and reliability issues even after extensive staff training.

#### Missed appointments.

Many centers had problems with missed appointments and late arrivals. To minimize the frequency of this drain on staff time, centers used a number of strategies. Some called participants several days or hours before to confirm an appointment; others tied research appointments to clinical appointments, which participants seemed more likely to keep. Staff flexibility was required to ensure that even missed assessments could be completed.

#### Lack of transportation.

Particularly for studies with low-income participants, transportation was a barrier to successfully completing assessments. A number of centers either paid for taxi services or reimbursed participants for alternate travel costs. One center transported participants to the office for an assessment after completing a home visit. Another center purchased and outfitted an RV that could be driven to participants’ homes and used as a roving assessment room. A number of centers purchased a car for the study to reduce mileage reimbursement costs and wear and tear on staff cars.

#### Issues of literacy, language, and culture.

Many centers have enrolled participants with limited education and low literacy. The sixth-to eighth-grade reading level that is standard for questionnaires was often too high for center participants. Wording and phrasing had to be simplified for all study documents, including consent forms, and most study instruments, including those designed for self-administration, had to be administered orally.

With very few tools validated on non-English speakers, centers have devoted considerable resources to translating materials. This posed unique challenges. Centers with Spanish-speaking participants have learned that Spanish-language instruments do not necessarily reflect the dialect spoken by participants. Languages like Hmong are largely oral, with a written form having only developed recently. Potentially embarrassing topics that evade translation—for example, specific birth control methods—must sometimes be described graphically. Few neurodevelopmental assessment tools exist in other languages (even in Spanish), and these are often only translated and not validated.

Centers also faced unexpected challenges related to the culture and acculturation of participants. Obstacles encountered by centers included participants not knowing or (with undocumented immigrants) not sharing their exact date of birth, being hesitant to provide biologic samples (because of concerns that those in possession of the sample have the power to hurt them), and reporting pregnancy relatively late in gestation (when the fetus was believed strong enough to withstand evil spirits). Focus groups with community members were instrumental in understanding these types of issues and planning the research accordingly.

#### Staffing issues.

Many centers have found that building trusting relationships with participants is best accomplished by hiring bilingual, bicultural staff who are from the local community and are assigned to follow particular families ideally from pregnancy through the child assessments. Although this is helpful in building trusting relationships, it can introduce systematic bias. Center studies require staff with a particular gift for engaging children and encouraging optimal performance. In addition, they must have an appropriate level of acculturation, bilingual fluency, education, and computer skills. Often, more in-depth training on data collection techniques is needed than when hiring from within the academic community.

Some center staff have found their work to be emotionally demanding because of the difficult circumstances of participants. In response, one center provided an opportunity for staff to meet with a social worker who specialized in Latino mental health issues. In addition, some staff members have been trained on community resources (e.g., food banks) and, in some cases, initiate contact for participants. To maintain interest in the research, some centers also provided ongoing staff enrichment opportunities, including monthly discussion groups on topics such as child abuse, housing code violations, and child development.

In all centers, the number of staff required to maintain a birth cohort; to conduct weekday, weeknight, and weekend assessments; and to complete quality control tasks was much larger than projected. Staff time and funds were taxed by the need for extensive training and the necessity of sending staff members in pairs to dangerous neighborhoods. Gaps in funding were extremely detrimental to centers in that valuable staff could not be maintained and new staff required time-consuming training.

## Retention

Retention of participants has been a critical concern for all centers. In the three centers that have completed follow-up to age 2 years, attrition rates ranged from 15 to 26%. For all centers, participants lost to follow-up differed from continuing participants in some demographic characteristics, such as age, marital status, medical insurance status, and race or ethnicity.

The most common reason for attrition was the inability to locate participants, usually due to disconnected phones and/or frequent or unreported moves (the latter was particularly true for a center that enrolled primarily migrant farmworkers). Lost participants were reinstated in some studies if they returned to the area or resumed contact. Some centers excluded participants who repeatedly missed appointments. Other participants refused to continue because of fatigue, lack of interest, or a partner’s disapproval. In a few cases, attrition was due to infant deaths.

All centers have used incentives to improve retention rates. Incentive amounts per visit have ranged from $20 to $300 (averaging ~$50), with some centers increasing the amount over the course of enrollment. Most centers provide incentives in the form of gift certificates (e.g., to grocery stores) rather than cash so as to minimize security concerns. Several centers offer bonus incentives for certain activities, such as calling study staff when in labor, returning on a separate day to finish an assessment or provide an additional sample, or providing new contact information on moving.

Most centers also provide small gifts such as toys, baby blankets, and tote bags. One center held a raffle at the end of the 24-month visits for participants who remained in the study, and another center intends to have yearly raffles. Although some centers have been successful in soliciting donations of raffle or incentive items, incentives remain a major budget item for all. In addition, some centers have questioned whether certain types of incentives—for example, educational items—could serve as an intervention in families with few such resources.

### Overcoming barriers to retention.

To improve retention, the centers used a variety of strategies, including sending letters when phones were disconnected, using mail-forwarding services, sending research staff to the last known address, and using contacts (family and friends) to get updated information on the participants or to pass a message along. Some centers have used Internet-based “reverse look-up” sites to obtain addresses for participants who consistently do not answer phone calls; sending a letter to the address has had some success. Frequent contacts with the participants by phone or mail have also helped to reduce attrition. Most centers contact participants every 3–6 months. These contacts include birthday cards, brief telephone interviews about the child’s health (e.g., respiratory disease or injuries), or simple “check-ins” with the family to remind them of the next phases of the study. Because of a gap in funding, one center had nearly 2 years between contacts. This lapse resulted in considerable attrition and required significant costs and personnel time to locate the families.

In addition to phone calls and mailings, centers used other techniques to maintain communication and retain participants. One center organized a health fair for participants. Another distributed photograph key chains reminding participants to call if they moved and inserted a new photo of the child at each visit to promote its use. Other centers provided magnetized business cards for families’ refrigerators or distributed staff pager and cell phone numbers to encourage communication. One center has permitted participants who have moved from the study area or desire limited participation to complete phone interviews only or allow medical record review. This center has also made weeklong summer trips to complete assessments with participants who have moved to other areas of the state.

## Research Infrastructure

### Data and Specimen Management Systems

Computerized databases are an essential component of all centers’ participant tracking systems. Center databases contain basic information about participants (e.g., date of birth), information about visit events (e.g., event type and date completed), and detailed information about biologic and environmental samples (e.g., date collected, number and volume of aliquots). Centers use these systems to generate periodic reports (e.g., projected events for the coming month and volume of stored samples) and to check the completeness of final data sets.

### Specimen Repository

As previously reviewed, there are many issues to be considered with regard to laboratory specimens (Eskenazi et al. 2003; Holland et al. 2003; Schulte and Perera 1993). All centers collected a variety of biologic samples (Table 5) from participants and/or environmental samples (Table 4) from home environments. Collectively, the centers obtained urine, peripheral blood, cord blood, breast milk, meconium, vernix, saliva, hair, placental tissue, infant formula, indoor and outdoor air, and house dust. The centers have analyzed levels of numerous compounds in these biologic and environmental samples, such as pesticides, phthalates, mercury, lead, cotinine, polycyclic aromatic hydrocarbons (PAHs), PAH–DNA adducts, allergens, endotoxin, antioxidant micronutrients, cytokines, immunoglobulin E (IgE), cholinesterase, and thyroid hormones. Some centers are also analyzing biomarkers of susceptibility, for example, DNA polymorphisms. An important goal of each center was to maximize future use of stored samples. Most centers banked samples for future analyses, such as blood samples for later derivation of RNA and for genomics assays using high-throughput methods based on polymerase chain reaction, chip, and microarray technologies (Appendix 3 for banked samples and Table 5 for intended analyses).

To assure the quality of the specimens for current and future use, the centers developed protocols for collecting, shipping, processing, and banking samples. Pilot studies were conducted to determine the collection and storage conditions necessary for stability of certain compounds and their range of levels in the cohort. Study protocols included written instructions and standard operating procedures, methods for documentation of procedures using chain-of-custody forms and discrepancy reports, and databases to track the location and flow of samples. Protocols were developed for quality assurance and control procedures, for separating specimens into several aliquots to eliminate the need for repeated thawing and freezing, and for avoiding potential contamination of the specimen. As part of their quality control protocols, most centers included field blanks, spikes, and duplicates in their analytical batch of samples. Most centers created bar-coded labels for specimens. Labels included the participant’s unique, coded identifier, the sample type, and the aliquot. In some cases, pilot studies were conducted to determine whether labels would withstand shipping and laboratory conditions over time. All these protocols aimed to maximize the potential for future use of sometimes low-volume samples (e.g., child blood samples).

#### Examples of problems in sample collection.

Blood collection from children is a challenge. Most centers collected research blood samples at the same time as clinical samples. This helped to avoid participant concerns about taking blood from children and pregnant women, especially in certain cultural groups. Researchers found it helpful to consult with community physicians to determine the amount of blood collection that is both clinically and culturally acceptable to the target population.

Centers found that collecting breast milk samples soon after delivery, although most convenient for the research team, was challenging for mothers. For most, the milk supply had not yet fully developed, and some new mothers (particularly primiparas) found it difficult to provide samples with a breast pump. In addition, some mothers feared that milk was being taken away from the baby. Later collection of breast milk avoided some of these problems, but timing problems arose for other sample types as well. When sample collections could not occur during a scheduled visit, centers scheduled extra visits or made alternate plans. For example, when children could not provide a urine sample, one center gave parents the supplies and instructions to collect the sample at home and arranged to pick it up the next day.

Studies conducted in rural areas faced additional barriers to successful collection and processing of samples. Centers with rural study sites encountered limited laboratory facilities that were not adequately equipped to process samples (e.g., to separate whole blood into blood products). For these centers, it was necessary to transport samples over long distances, increasing costs. In locations where necessary goods and services (e.g., dry ice or courier services) were in short supply, it was also difficult to ensure the prompt stabilization of samples. Finally, some rural areas lacked skilled pediatric phlebotomists.

## Ethical Issues

The Children’s Centers have found themselves operating in a time of increasing ethical complexity. Particularly since the implementation of HIPAA, it has become more time-consuming to obtain participants’ informed consent. Concerns about potential lawsuits have increased and been exacerbated by the Grimes vs. Kennedy Krieger case (Mastroianni and Kahn 2002). Finally, centers struggle with conflicting ethical issues, such as deciding when the health and safety of a child takes precedence over a promise of confidentiality.

### Consent and assent.

Longitudinal studies demand lengthy and complex consent forms. Ensuring that participants are well informed has been challenging for the centers and has required the allocation of adequate time to inform participants about the study and to review the consent form. For centers using medical records, the completion of HIPAA subject authorization forms adds time to the consent process. Centers’ consent forms differ in level of complexity and in time needed to complete them.

Centers have found it important to inculcate in staff an understanding that consent is an ongoing process. Instead of training staff to simply procure participant signatures, centers have trained staff to solicit and answer participants’ questions so that they can make informed decisions.

All centers recognized the importance of writing consent forms at a reading level understandable to all. Some centers wrote consent forms at an eighth-grade level, whereas others felt that even sixth-grade level was too high to assure comprehension. In addition to providing consent forms in multiple languages, some centers read consents aloud to participants to ensure that everyone, including participants who are embarrassed to admit their low literacy level, fully understood the information. Some centers solicited feedback from community partners, community board members, and community-based staff (in addition to the IRB) to help ensure that appropriate language was used. The centers’ experiences suggest that the language and style of a consent form in one community may not be appropriate in others.

Some studies used additional measures to enhance understanding of consent forms. Several centers used timetables and schedules to communicate study procedures or provided lists outlining the important items on the consent. One developed a short checklist to verify that participants understood the key aspects of the study. Two centers divided consent between two documents, one covering enrollment through delivery, and one covering the period after birth. This decreased the amount of complex information that participants had to digest at each visit, and gave participants an opportunity to re-evaluate their participation at a midway point. However, some participants expressed frustration with the continuing requests, indicating they would prefer full disclosure of the protocol up front.

Centers gave careful thought to who must consent to participate at each stage of the research. In all cases, pregnant women or mothers were asked to consent to their own participation and that of her child. However, once children reached a certain age (generally 5–9 years), child assent was usually also required by the IRB, posing new challenges for the centers. Centers needed to clarify for themselves and for their staff the difference between encouraging a child to try a new task and coercing him or her to do so. Some centers also needed to consider consent procedures in cases when the mother no longer had custody of the child (either officially or unofficially). Finally, centers that conducted home visits considered whether it was adequate for the mother to consent to a visit in a home shared with other families. In some such cases, centers skipped home visits to these participants or limited the visit to the portion of the home in which the participating mother and child lived.

### Banked samples and informed consent.

Many centers have banked samples for future studies. This process requires special consideration, in that participants must be informed about and consent to future uses of these samples. Several centers’ consent forms allowed participants the option of either not having samples banked or not allowing future analysis of samples for unrelated studies. At least one center has needed IRB reapproval for each new analysis of banked samples. The center at the University of Washington has participated in a consortium formed by the Centers for Disease Control and Prevention (CDC) to develop informed consent approaches for integrating genetic variation studies into population-based research (Beskow et al. 2001); the group developed an informed consent template (CDC 2001).

### Confidentiality and consideration of children at risk.

All centers strove to protect the identity and personal information of all participants. Some centers found it challenging, however, to maintain confidentiality in small or close-knit communities, especially when the research staff was hired from the local community. Most centers instructed staff on when to remove themselves from assessments with participants they know personally and on how to interact with participants when they meet in other settings.

Centers were also vigilant to ensure confidentiality within computerized databases. Centers modeled their own data management systems around guidance provided by their IRBs. All computerized files were password protected with knowledge of passwords restricted to a small number of staff, and the number of computer or paper files containing both the participant study number and identifying information (e.g., name) was limited. In complex studies with multiple contacts, centers found it necessary to work with both the IRB and the research staff to identify the types of linked information necessary for day-to-day operations and to provide that information with the least possible risk to participants. Centers kept files linking study numbers with participant name separate from event and sample tracking databases, linking them briefly only when necessary (e.g., to generate mailing lists of participants missing a particular event).

Certificates of confidentiality, which protect identifiable research information from forced disclosure, including in the case of legal action, are an important component in protecting participant confidentiality. However, many centers faced or anticipated facing circumstances in which they would need to break the promise of confidentiality without participant consent, for example, in cases where child abuse, severe depression, drug use or traffic in the home, and other potentially dangerous conditions were observed. Some centers have elected not to report housing code violations that do not pose an immediate threat to the child, because there is no clear legal mandate or options for the families, and because participants may fear eviction or reprisals from landlords. Centers developed clear protocols that included provisions for referral or reporting, including lists for center staff of what constituted an immediate housing threat. Staff were trained on human subject’s protection requirements and child abuse and neglect reporting issues, including mandatory or discretionary reporting protocols. Because some variation exists in state laws regarding mandated reporting of child abuse, the centers’ experiences suggest that special care should be taken in planning a nationwide study to ensure that protocols are in compliance with both the specific state laws and IRB requirements. Disclosure of such requirements (e.g., the need to report child abuse) was incorporated into consent forms, despite concern that it would repel some participants.

Centers have also developed protocols on intervening in cases of clear developmental delays or undiagnosed physical health problems. Most protocols include timely screening of developmental assessments and questionnaires to ensure prompt referral or treatment. Another aspect of these protocols is the centers’ designated cutoff score for developmental tests (e.g., > 2–3 standard deviations below the mean), children scoring below which are referred with parental permission for further evaluation or intervention. To ensure adequate follow-up of identified children, centers found it useful to identify local resources beforehand; the number of such resources, of course, varied by community. Centers were also required to report some exposure measures, such as lead results, to public health authorities when they exceeded certain action levels.

## Communication

Communicating study results is a key step in any research project. In addition to publishing results in scientific journals, centers sought to share findings with participants and community members. Centers elicited the guidance of community collaborators to decide when and how to disseminate results, including how to craft messages that would be clearly understood by and of interest to the community. In some cases, communities expected interventions and actions that were outside the scope of the research; to prevent false expectations, the centers agree it is important to communicate the purposes and limitations of the research beforehand.

### Timing of results communication.

Particularly in longitudinal studies with distant visit points that employ new or experimental laboratory methods, there is often a long delay between when data collection begins and when results are communicated to participants and communities. To ensure themselves adequate time to analyze and interpret results without causing undue frustration in participants, most centers found it beneficial to disclose all anticipated delays during the informed consent process.

Many centers have made it a priority to disclose findings to participants and/or community advisory boards before their publication in journal or newspaper articles. This disclosure has been an important step in building trust between researchers, participants, and communities. Community members resent hearing findings for the first time from the media.

### Communication tools.

Dissemination strategies developed in collaboration with community advisory boards have included newsletters, fact sheets, pamphlets, press releases in local papers, pay-stub inserts, radio programs (particularly useful in rural areas), town hall meetings, and Internet sites. One center has a monthly radio program in which they report study progress and present a health and safety message. Investigators at all centers share their findings with other scientists and the public via presentations at national conferences, publications in peer-reviewed scientific journals, lectures at colleges and universities, and presentations at community meetings. Ideally, centers would also like to communicate results to children. The center at the University of Washington, based on results from their study of pesticide exposure in children, has created coloring books and curricula to educate preschool and school-age children on how to prevent such exposure.

Specialized tools are often needed for studies that target low-literacy or non-English-speaking communities. Many centers publish information in more than one language, and some centers have attempted to develop pictorial rather than verbal messages.

### Group-versus individual-level results.

Perhaps the biggest communication issue that the centers have faced has been whether to provide individual-level results, particularly for measures of exposure or internal dose. The argument in favor of providing such results is that participants have the right to know; the counterargument is that participants may be unnecessarily alarmed by results with no interpretable meaning. Generally, results with a clear clinical implication (e.g., blood lead levels) have been reported to participants, whereas results without clear clinical implications (e.g., urinary pesticide metabolite levels) have not been returned. One center, however, on the basis of community advisory board input, has decided to offer participants the option of requesting their individual pesticide levels. That center is currently in the process of developing materials to provide these results and will work closely with community health care providers when clinical questions arise.

Regardless of whether group or individual-level results are returned, the centers agree it is important to provide to participants a context for these results. Providing a comparison, either to other study participants or nationwide data, has been particularly helpful. In communicating results, centers aim to clearly describe their implications for health and well-being; when these implications are not known (as in the case of pesticides), centers state this honestly (Faustman et al. 2000).

## Conclusion: Lessons Learned

In anticipation of the National Children’s Study, lessons can be learned from the birth cohort studies being conducted by the National Institute of Environmental Health Sciences/U.S. Environmental Protection Agency Centers for Children’s Environmental Health and Disease Prevention Research. The National Children’s Study shares a mission with these centers—to understand the environmental causes of developmental diseases—and thus can benefit from the lessons learned during their implementation. Collectively, the centers offer the following advice gleaned from 7 years of research:

- Building community infrastructure and trust is essential, especially in populations that are difficult to reach. Formative research, including focus groups with community members, is useful in understanding cultural barriers.
- Research goals must be prioritized. Participant fatigue limits the length of the research protocol. Hence, the research needs of a large team of investigators need to be negotiated and prioritized. These research priorities may vary by geographic location.
- A research study should be self-sufficient and rely minimally on clinical staff. Tagging a research protocol onto a clinical visit or hospitalization is usually not feasible. Medical care facilities, in particular those that treat low-income populations, are already overburdened.
- Research protocols must be flexible and allow for variations in levels of care and medical protocols in hospital and clinics. Usually, only large urban areas have academic tertiary care facilities. If multiple facilities in both rural and urban areas are involved in the research, protocols must be flexible.
- Long-term, continuous employment of high-quality and flexible research staff is imperative for the success of the study. Retaining high-quality staff over the course of the study is the key to project success. All centers greatly underestimated the staffing needed to enroll, evaluate, and maintain the birth cohort.
- It is essential to find space that is without distraction and convenient to the families. This is difficult to find in medical care settings or in the home. Providing participant transportation was costly, but it was essential for keeping centers’ families enrolled.
- Research protocols should be piloted and documented, and quality control protocols should be developed and enforced. Although the development of quality control protocols is standard for laboratory research, frequent checks and close oversight are also necessary for neurodevelopmental assessment and interviewing. All centers felt that the time and resources necessary for this effort were underestimated.
- It may be necessary to allow for variations in the type of information collected and the methods of collection to allow for differences in literacy, language, and culture across disparate populations participating in a national study.
- Biologic and environmental specimens should be carefully collected, processed, and banked in multiple aliquots. Specimen collection may need to vary by site to accommodate cultural concerns and logistical differences. There should be foresight in the funding of a biorepository for future generations of researchers.
- Longitudinal cohort studies must closely track participants. Tracking families may be difficult, especially with mobile populations. Efforts should be made to maintain frequent contact with participants and to collect the contact information of people who will know their whereabouts. Again, the quality of the staff and their relationship to the participants is essential to retaining the cohort.
- It is necessary to allow adequate time to obtain informed consent. Obtaining informed consent for low-literacy and immigrant populations may require additional steps. Consents should be written to allow materials and specimens to be used for future studies.
- The complex ethical issues involved in conducting a birth cohort study, especially in low-income populations, must be carefully considered. When institutional IRBs differ, deference should be given to an IRB familiar with the culture of the population. Given the changing research climate, observational studies without a component of prevention or intervention may be perceived as unethical in the future.
- Study results must be communicated to participants and lay and scientific communities in a timely and sensitive manner. A communication plan needs to be developed with community partners. The cost of regular communication with the community must be factored into the research plan.
- If multiple centers are involved in the research, it is essential that there is close and regular communication among them aimed at problem solving and assuring similar methodologies. Resources should allow for frequent and ongoing communication.
- Funding for a longitudinal birth cohort study must be adequate for the start-up period and continuous, without gaps, through the long term. Opportunities to evaluate specific developmental milestones may be lost when there are funding lapses. Long breaks between participant contacts can greatly increase attrition rates of valuable populations. The necessary start-up time, including time for formative research, was greatly underestimated by all centers.

On average, the centers have allocated at least $500,000 per year to their birth cohort studies. Given that the average sample size for these birth cohorts is 500, this would translate into a cost of about $500 million for the first 5 years of study of the 100,000-person birth cohort proposed for the National Children’s Study. This does not include additional costs such as for coordination among centers and long-term storage of specimens.

Despite numerous logistical challenges in collecting longitudinal birth cohort data, the Centers for Children’s Environmental Health and Disease Prevention Research have been successful in enrolling and maintaining a variety of populations, including from minority and low-income communities. Although the challenges of longitudinal data collection may be greatest in communities with the poorest and most marginalized populations, we maintain that it is crucial to include these diverse populations from both rural and urban environments to understand the health of those children at greatest risk for environmental hazards (Metzger et al. 1995; Pirkle et al. 1996; Sarpong et al. 1996; Whyatt et al. 2002). We hope that the lessons learned from the Centers for Children’s Environmental Health and Disease Prevention Research can help to inform the National Children’s Study.

## Figures and Tables

**Table 1:** Study overview.

Component	University of Illinois	University of California, Berkeley	Columbia University	Mount Sinai Medical Center	Cincinnati Children’s Hospital
Study design	Prepregnancy/pregnancy cohort	Pregnancy/birth cohort	Pregnancy/birth cohort	Pregnancy/birth cohort	Pregnancy/birth cohort
No. of subjects enrolled	164 couples^a^ (goal = 400)	601 women	556 women^a^ (goal = 730)	479 women	300 women^a^ (goal = 400)
Characterization of the population	Hmong and Laotian couples of childbearing age	Low-income Mexican and Mexican-American women in agricultural community	Dominican and African-American women	Public and private prenatal patients	Public and private prenatal patients
Location	Green Bay and Appleton, Wisconsin	Salinas Valley, California	New York City (Harlem, Washington Heights, South Bronx)	New York City (East Harlem)	Cincinnati, Ohio
Major exposures	PCBs, methylmercury	Pesticides, allergens, metals	PM, DEP, PAH, ETS (cotinine), pesticides, allergens, metals	Pesticides, PCBs, metals	Metals, PCBs, pesticides, tobacco smoke (cotinine), alcohol
Major outcomes	Growth, hearing, neurodevelopment/behavior	Growth, neurodevelopment/behavior, biomarkers, asthma/respiratory disease	Growth, neurodevelopment/behavior, biomarkers, asthma/respiratory disease	Growth, neurodevelopment/behavior, biomarkers	Growth, neurodevelopment/behavior, hearing, asthma/respiratory disease

Abbreviations: DEP, diesel exhaust particulates; ETS, environmental tobacco smoke; PAH, polycyclic aromatic hydrocarbon; PCBs, polychlorinated biphenyls; PM, particulate matter.

a Still enrolling subjects.

**Table 2:** Eligibility criteria.^a^

Criterion	University of Illinois	University of California, Berkeley	Columbia University	Mount Sinai Medical Center	Cincinnati Children’s Hospital
Age (years)	Women 18–44, Men ≥ 18	≥ 18	18–35	≥ 16	≥ 18
Language/ethnicity	Hmong, Lao	English, Spanish	African-American, Dominican	English, Spanish	English
Gestational age (weeks)		< 20		≤ 26	13–19
Exclusions	Will exclude infants after delivery if no prenatal samples	None	Diabetes Hypertension HIV Smoker Illicit drug use in last year First prenatal visit > 20 weeks gestation Infants after delivery if no prenatal interview, personal monitoring data, or delivery blood Lived in study area for < 1 year before pregnancy	Multiparas Multiple gestations Alcohol use Illicit drug use Infants after delivery if < 32 weeks gestation, < 1,500 g, birth defect, or no prenatal specimens	Diabetes Seizure disorder HIV Schizophrenia Bipolar disorder Thyroid disease Living in mobile home Home built before 1979
Prenatal care at participating clinic	No	Yes	Yes	Yes	Yes
Planned delivery at participating hospital	No	Yes	Yes	Yes	Yes
No plans to move			< 1 year after delivery		
Other	Residents of Green Bay/Appleton	MediCal eligible			
Who determined eligibility?	Research staff	Clinic staff	Research staff	Research staff	Research staff

a Blank fields indicate no relevant eligibility criteria for that center.

**Table 3:** Contact points and types.

Contact	University of Illinois	University of California, Berkeley	Columbia University	Mount Sinai Medical Center	Cincinnati Children’s Hospital
Questionnaire	Enrollment, every 2 months before pregnancy, monthly during pregnancy, delivery, child age 6, 9, 12 months	Pregnancy (enrollment mean = 13 weeks), 3rd trimester, delivery (mother and father), child age 6, 12, 24, 42, 60,^a^ 84^a^ months	Pregnancy (3rd trimester) child age 6, 12, 24, 36, 60, 72,^a^ 84^a^ months	Pregnancy (3rd trimester), child age 12, 24, 48,^a^ 72,^a^ 84^a^ months	Pregnancy (enrollment mean = 20 weeks), child age 4 weeks, 12, 24 months; injury questionnaires every 3 months; sleep questionnaire every 6 months
Home walk-through		Pregnancy, 6, 12, 24, 42, 60^a^ months	Pregnancy, 12, 36, 60 months		Pregnancy, 12, 24 months
Neurodevelopment assessment	Birth, 6, 9,12 months	Birth, 6, 12, 24, 42, 60^a^ months	6, 12, 24, 36, 60, 84^a^ months	Birth 12, 24, 48,^a^ 72,^a^ 84^a^ months	Birth, 4 weeks, 12, 24 months
School evaluation		84^a^ months	96^a^ months		
Growth assessment	Birth, 6, 9,12 months	Birth, 6, 12, 24, 42, 60^a^ months	Birth, 6, 12, 24, 36, 60 months	Birth, 12, 24, 48,^a^ 72,^a^ 84^a^ months	Birth, 4 weeks, 12, 24 months
Respiratory assessment		6, 12, 24, 42, 60,^a^ 84^a^ months (by questionnaire); spirometry at 60 months	Every 3 months from birth to 24 months; every 6 months from 24 to 60 and to 84^a^ months (by questionnaire); spirometry at 60 months		
Incentives	$20–35 in gift certificates per prepregnancy and pregnancy visits; amounts for child visits are to be determined; T-shirts, water bottles, fish measuring tapes, back-to-school packets; delivery gift baskets with baby T-shirts, socks, bottles; fishing supply raffles	$20–60 in gift certificates per visit; car seat or stroller at delivery; hats, T-shirts, tote bags, toys; raffle after 24 and 60^a^ months	$50–300 in cash per visit, educational toys	$50 in cash per visit, toys	$25–100 in gift certificates per visit; tote bags, T-shirts, baby blankets, age-appropriate books

a Planned for second 5-year funding cycle.

**Appendix 1:** Questionnaire information collected.

Factors	University of Illinois	University of California, Berkeley	Columbia University	Mount Sinai Medical Center	Cincinnati Children’s Hospital
Demographic information					
Demographics	X	X	X	X	X
Occupation	X	X	X	X	X
Household income	X	X	X		X
Health and development					
Reproductive history	X	X	X	X	X
Medical history	X	X	X	X	X
Medication use	X	X	X	X	X
Child sleep	X				X
Breast-feeding/child diet	X	X	X	X	X
Developmental milestones	X	X		X	
Respiratory symptoms, illness		X	X	X	X
Exposure assessment					
Housing characteristics	X	X	X	X	X
Pesticide exposure	X	X	X	X	X
Allergen exposure		X	X		X
House cleaning habits		X		X	
Injury hazards					X
Home remedies	X	X	X	X	
ETS	X	X	X	X	X
Household members	X	X	X	X	X
Household pets		X	X	X	X
Fish consumption	X		X	X	X
Social factors					
Social support	X	X			X
Maternal depression	X	X			X
Psychological distress			X		
Parenting stress	X				X
Marital conflict			X		
Life events		X			
Quality of life			X		
Neighborhood quality			X		X
Sense of control					X
Neighborhood cohesion					X
Family resources					X
Material hardship		X	X		X
Acculturation	X	X			
Immigration history	X	X			X
Childcare	X	X	X	X	X

ETS, environmental tobacco smoke.

**Appendix 2:** Home visit information collected.

Characteristic	University of California, Berkeley	Columbia University	Cincinnati Children’s Hospital
Age of housing			X
GPS coordinates	X	X	X
Type and condition of flooring	X		X
Cockroaches	X	X	X
Rodents	X	X	X
Mold/mildew	X	X	X
Wall moisture	X	X	X
Water damage	X	X	X
Peeling paint	X	X	X
Pets	X	X	X
Proximity to agricultural fields	X		
Proximity to major streets	X	X	
Pesticide use	X	X	X
Pesticide storage	X		
Gas stove/gas heater	X	X	X
Cleanliness	X		X
Safety of home environment	X		X
Lead hazards			X

**Table 4:** Environmental samples.

Sample	University of California, Berkeley	Columbia University	Cincinnati Children’s Hospital
House dust	Pregnancy, 6, 12 months	Pregnancy, 12, 36, 60 months	Pregnancy, 12, 24 months
Lead			X
Pesticides	X		X
Fungal spores/pollen	X	X	
Allergens/endotoxin	X	X	X
Vehicle dust	6 months (subset)		
Pesticides	X		
Burkard air sampling, house	Pregnancy, 6, 12 months		
Fungal spores/pollen	X		
Burkard air sampling, area	Ongoing	12 months	
Water			Pregnancy, 12, 24 months
Soil			Pregnancy, 12, 24 months
Personal air sampling		Pregnancy	
Infant formula			1 month
PCBs			X
Lead			X
Pesticides			X
Phthalates			X

PCBs, polychlorinated biphenyls.

**Table 5:** Biologic samples—maternal, paternal, and child (and attendant analyses).

Sample	University of Illinois	University of California, Berkeley	Columbia University	Mount Sinai Medical Center	Cincinnati Children’s Hospital
Maternal blood	Enrollment, 1st, 2nd trimester, delivery, 6 weeks postpartum (pesticides, lead, other metal, PCBs, thyroid hormone)	2nd, 3rd trimester, delivery (pesticides, lead, PCBs, IgE, cholinesterase, genetic polymorphism, thyroid hormone)	1 day postpartum (pesticides, lead, mercury, tobacco, PCBs, IgE, DNA adducts, genetic polymorphism, micronutrients)	3rd trimester (pesticides, lead, PCBs, cholinesterase, genetic polymorphisms)	16, 26 weeks gestation, delivery (pesticides, lead, mercury, tobacco, genetic polymorphism)
Maternal urine	Monthly during menstrual cycle (subset) (phthalates, hCG)	Enrollment, 2nd, 3rd trimester, delivery 6 months postpartum (subset) (pesticides)	3rd trimester, every 2 weeks < 34 weeks gestation, delivery (subset) (pesticides)	3rd trimester (pesticides)	16, 26 weeks gestation, delivery (pesticides, phthalates)
Placental tissue			Delivery		
Breast milk		Delivery, 6 months postpartum (pesticides)			1 month postpartum (PCBs, lead, pesticides, tobacco, phthalates)
Maternal saliva					16 weeks postpartum (pesticides)
Maternal hair					Enrollment, 2nd trimester, 4 weeks postpartum (tobacco)
Paternal blood	Enrollment (PCBs)				
Paternal urine		Delivery (pesticides)			
Cord blood	Delivery (pesticides, mercury, PCBs, chromosome damage)	Delivery (pesticides, lead, IgE, cholinesterase, genetic polymorphisms)	Delivery (pesticides, lead, mercury, tobacco, PCBs, IgE, DNA adducts, genetic polymorphisms, micronutrients)	Delivery (lead, cholinesterase, genetic polymorphisms)	Delivery (pesticides, lead, mercury, iron, tobacco, PCBs, genetic polymorphisms)
Child blood	Planned (lead)	12, 24, 60^a^ months (lead, IgE, cholinesterase, genetic polymorphisms, cytokines)	24, 36, 60 months (pesticides, tobacco, IgE, cytokines)		12, 24 months (pesticides, lead, mercury, iron, tobacco, PCBs, genetic polymorphisms)
Child urine		6, 12, 24, 42, 60^a^ months (pesticides)	36, 60 months (pesticides)	12, 24 months (pesticides)	12, 24 months (pesticides, phthalates)
Child meconium			Birth (pesticides)		Birth (pesticides, lead, mercury, tobacco, PCBs, alcohol)
Child vernix					Birth (pesticides, tobacco)
Child saliva		42, 60^a^ months (pesticides)			
Child hair					12, 24 months (tobacco)

Abbreviations: hCG, human chorionic gonadotropin; PCBs, polychlorinated biphenyls.

a Currently under way as part of second 5-year funding cycle.

**Appendix 3:** Banked blood samples.

	University of California, Berkeley		Columbia University		Mount Sinai Medical Center		Cincinnati Children’s Hospital	
Sample type	C	M	C	M	C	M	C	M
Whole blood	X	X	X	X	X	X	X	X
Serum	X	X			X	X	X	X
Clot	X	X						
Plasma	X	X	X	X	X	X		
Buffy coat	X	X	X	X		X		
Red blood cells	X	X	X	X		X		
Lymphocytes (cryopreserved)	X		X	X				
Blood smears	X	X						
Cholinesterase (stabilized)	X	X						
DNA	X	X	X	X	X	X	X	X
Other specimens	X	X	X	X			X	X

Abbreviations: C, child/cord blood; M, mother.
